# Anesthetic Management of a Pregnant Patient With a Large Anterior Mediastinal Mass Undergoing Diagnostic Bronchoscopy: A Case Report

**DOI:** 10.1155/cria/2053701

**Published:** 2025-10-23

**Authors:** Rei Ndoni, Aariya Srinivasan, Ryan Dougherty, Colin Nahrstedt, Jia Liu, Sabry Ayad

**Affiliations:** ^1^ Department of Anesthesiology Research, Cleveland Clinic Foundation, Cleveland, Ohio, USA, clevelandclinic.org; ^2^ Staff Anesthesiologist, Cleveland Clinic, Fairview Hospital, Cleveland, Ohio, USA, clevelandclinic.org

## Abstract

This case report details the successful use of awake fiberoptic intubation in a 28‐year‐old pregnant patient at 21 weeks gestation, who presented for diagnostic mediastinoscopy with biopsy secondary to a large anterior mediastinal mass suspected to be lymphoma. MRI displayed encasement of the superior mediastinal vasculature and 13.0 cm × 7.6 cm coalescence in the anterior mediastinum. Given the size of the mass and potential for airway and vascular complications, awake fiberoptic intubation was chosen to ensure safety for both mother and fetus. The case underscores the efficacy of awake fiberoptic intubation in complex airway management scenarios in pregnant patients, highlighting the importance of a multidisciplinary approach to optimize patient care and outcomes.

## 1. Introduction

The anterior mediastinum is an area in the chest that contains important structures including the heart, lymph nodes, thymus, trachea, and esophagus. Masses may arise in this area due to many diseases such as lymphomas, thymomas, germ cell tumors, and thyroid goiters. An anterior mediastinal mass (AMM) in a pregnant woman can present unique challenges due to the anatomical changes associated with both the AMM and pregnancy as well as concerns about fetal well‐being. This report details the periprocedural considerations and anesthetic management undertaken that illustrate the complexities of airway management in this unique clinical scenario. The successful outcome underscores the importance of multidisciplinary collaboration in the management of patients with significant mediastinal disease during pregnancy.

## 2. Case History and Presentation

The patient is a 28‐year‐old female, G1P0 at 21 weeks pregnant, who presented for diagnostic cervical mediastinoscopy. She has a past medical history of anxiety and postural orthostatic tachycardia syndrome and past surgical history of encephalocele repair at birth. A Maternit21 Plus (Laboratory Corporation of America) noninvasive pregnancy test was performed which reported abnormal data for multiple chromosomes. Due to this result and concern for underlying malignancy, genetic counseling and hematology were consulted. Patient was subsequently referred to the NIH incidental detection of maternal neoplasia through noninvasive cell‐free DNA analysis (IDENTIFY) study where an MRI revealed she had diffuse enlarged abdominal and supraclavicular lymph nodes concerning for stage 3 lymphoma. She was subsequently referred to thoracic surgery and recommended to undergo diagnostic mediastinoscopy with biopsy.

### 2.1. Preoperative Airway Assessment

Airway examination revealed a Mallampati classification of II, adequate mouth opening, and normal thyromental distance. The patient had a short neck but normal neck mobility. There were no loose or prominent teeth, and she had no history of snoring or obstructive sleep apnea. She also denied prior head and neck radiation or surgery. Her BMI was 30.4 kg/m^2^. Given the presence of a large AMM and gravid uterus at 21 weeks, an awake fiberoptic intubation (AFOI) was planned to maintain spontaneous ventilation and avoid airway compromise during induction.

### 2.2. Investigation and Diagnosis

**Figure 1:**
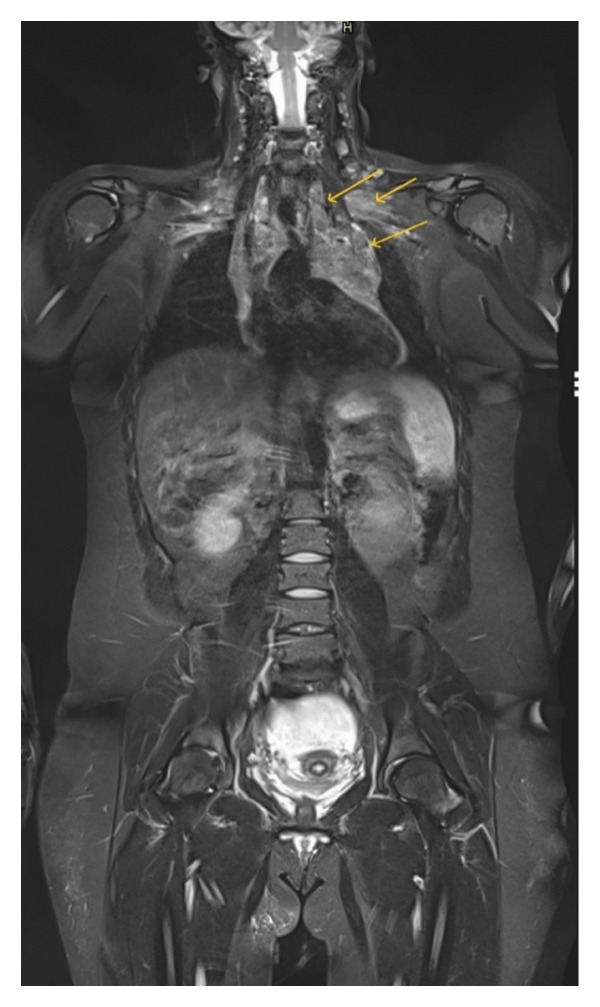
Coronal view short tau inversion recovery (STIR) sequence. Coalescent adenopathy measuring 13.0 cm × 7.6 cm in the anterior mediastinum and cervical adenopathy.

**Figure 2:**
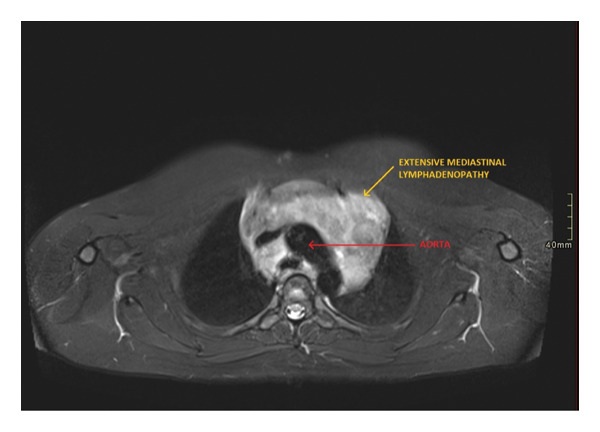
Axial view short tau inversion recovery (STIR) sequence. The superior mediastinal vascular structures encased by adenopathy.

**Figure 3:**
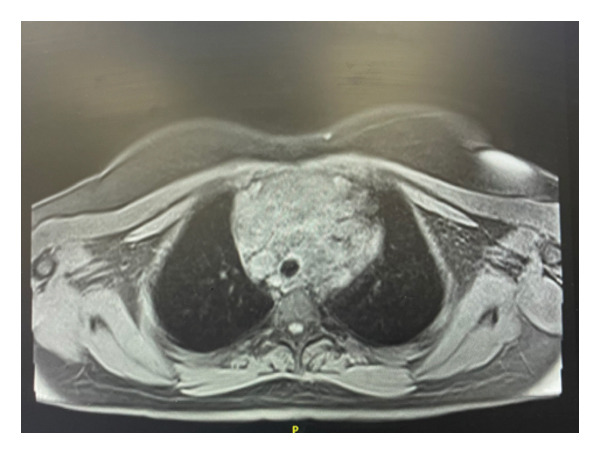
Axial view STIR sequence showing the mass over the carina.

Whole body MRI was performed during NIH evaluation. MRI study revealed adenopathy of the supraclavicular, internal mammary, and mediastinum concerning for lymphoma. In the supraclavicular region, adenopathy of 4.0 cm × 2.0 cm was noted (Figure 1). In the mediastinum, adenopathy was noted to encase the superior mediastinal vascular structures (Figure 2). Lymph nodes in the mediastinal region displayed extensive adenopathy with a coalescence in the anterior mediastinum measuring 13.0 cm × 7.6 cm (Figure 3). Additionally, the internal mammary adenopathy measured 2.1 cm × 1.3 cm. In the abdomen, nodules were noted in the right lobe of the liver measuring 1.9 cm × 1.3 cm and hepatoduodenal ligament nodule measuring 3.0 cm × 1.5 cm.

Presurgical ECHO was performed due to expectation for patient to receive cardiotoxic agents as part of her therapy for suspected lymphoma. ECHO reported an ejection fraction of 60% without significant valvular abnormalities or changes in ventricular size and function. Midascending aorta was measured at 2.9 cm, and inferior vena cava was measured at 1.4 cm.

### 2.3. Management

Due to the potential for airway and hemodynamic complications related to both pregnancy and the mediastinal adenopathy, the decision was made to proceed with an AFOI. Extensive discussion with the patient regarding airway, sedation, and the management of the pregnancy in the event of advanced cardiac life support necessitating chest compressions occurred. The patient was agreeable with the anesthetic plan and understood the risks of aspiration, airway and vascular compression from the AMM, and potential issues with the pregnancy in the event of ACLS. The obstetric team was consulted for fetal monitoring. Due to the fetus being in the previable period, only preoperative and postoperative fetal Dopplers were obtained. Vital signs in the preoperative area were a maternal heart rate of 85 bpm, blood pressure of 115/73 mmHg, and oxygen saturation of 100% in room air. A preintubation arterial line was placed for hemodynamic monitoring. Bispectral Index (BIS) monitoring was employed throughout the procedure to guide the depth of anesthesia. Values were maintained between 30 and 60, which were considered adequate to minimize intraoperative awareness while avoiding excessive sedation. 50 mcg fentanyl was given following arterial line placement for preprocedure comfort. 0.2 mg glycopyrrolate IV was given 15 min prior to intubation to reduce oral secretions. A three‐part strategy was utilized for topical anesthesia prior to attempting intubation. Nebulized lidocaine was started in the preoperative area, viscous lidocaine was applied to an oral airway, and 4% lidocaine was applied directly to the vocal cords through laryngotracheal anesthesia (LTA). Lidocaine is sprayed in a fine mist or jet directly onto the cords and surrounding mucosa using a sterile anatomically curved plastic cannula with attached vial injector. The patient was placed in beach chair position with left uterine displacement and preoxygenated in the sniffing position with the anesthesia circuit. A flexible bronchoscope was used to introduce a 7.0 mm endotracheal tube. Following airway securement, general anesthesia was induced with intravenous fentanyl, propofol, and rocuronium. The case was conducted under total intravenous anesthesia (TIVA) using a propofol infusion to prevent postoperative nausea and vomiting (PONV). No airway or hemodynamic compromise occurred during sedation, intubation, or induction of anesthesia. If airway compromise occurred, the plan was to be prepared to turn the patient into the lateral positioning, or to try rigid bronchoscopy to stent open the airway. Due to limited data available on sugammadex use in pregnancy and potential for organogenesis defects seen in animal models, neuromuscular blockade was reversed with neostigmine and atropine [1]. The patient was extubated and had no postoperative complications.

### 2.4. Outcomes and Follow‐Up

Due to cellular damage during procurement of the sample, adequate flow cytometry analysis was unable to be performed. Patent subsequently underwent a flexible bronchoscopy and robotic assisted mediastinal mass biopsy 10 days after the initial surgery. Flow cytometry of second biopsy confirmed nodular sclerosis subtype of classic Hodgkin lymphoma.

## 3. Discussion

The management of a second‐trimester pregnant patient with a large AMM presents unique challenges for the anesthesiologist due to the potential for airway and vascular compromise compounded by the physiological changes of pregnancy. Preoperative airway assessments such as Mallampati and neck circumference have been shown to increase throughout pregnancy while the oropharyngeal junction becomes smaller. Additionally, fat infiltration and cephalad movement of the upper airway occur further obscuring visualization of airway structures. Decrease in functional residual capacity due to compression of the lungs secondary to elevation of the diaphragm is well known in pregnancy. Increases in anterior–posterior diameter of the ribs and chest wall from relaxin hormone secretion allow for accommodation of the growing uterus and protect against large alterations in lung volume [2]. With the significant changes occurring in this area during pregnancy, space occupying lesions become of greater concern.

The anatomy of the anterior mediastinum contains major cardiopulmonary structures, and masses in this area can easily lead to obstruction. Complications of AMMs include superior vena cava syndrome, pulmonary vascular obstruction, and upper or lower airway obstruction. Muscular relaxation secondary to general anesthesia places patients with masses in this area at significant risk of compression and subsequent cardiopulmonary collapse. Lighter planes of anesthesia or even local anesthesia may be considered depending on size and location of the mass. For those masses requiring general anesthesia, careful preprocedure evaluation with detailed imaging should be considered [3].

In the case presented above, the patient’s gravid state and location of the AMM in this case carries an increased risk of airway obstruction and hemodynamic instability beyond either pathology alone. Furthermore, induction of general anesthesia reduces lung volumes and increases dead space [3]. In the pregnant patient, this further alters and complicates the respiratory physiology. In this patient, careful consideration was given to airway management. AFOI allowed for the preservation of spontaneous ventilation, minimizing the risk of airway collapse during induction [4]. This strategy is consistent with recommendations in the literature for patients with AMM, which have shown that positive pressure ventilation or neuromuscular blockade may exacerbate airway obstruction by reducing airway support from external pressures [3]. Failure rates of AFOI are as low as 0%–2% [5].

Local topical anesthetic is commonly used for patient comfort and success in AFOI particularly when minimal sedation is given. Sedatives such as dexmedetomidine, midazolam, propofol, and remifentanil are commonly used adjuncts. Dexmedetomidine has been suggested to be somewhat safer than other sedatives during AFOI [6]. In the pregnant population, dexmedetomidine’s use has historically been limited due to birth defects in animal models and concerns placental transfer [7, 8]. Newer studies indicate that while placental transfer does occur, it may not have as significant clinical effects in the immediate peripartum period or neonate as once thought [7]. Propofol is a very commonly used intravenous anesthetic for ICU sedation, monitored anesthesia care, and induction of general anesthesia. Limited placental transfer and significantly lower fetal concentrations of propofol have been demonstrated in animal models [9]. Additionally, propofol does not carry an advisory from the FDA regarding its use in pregnant patients; however, infusions over 3 h during peak fetal brain development were specifically advised against [10].

Current practice guidelines from the American College of Obstetrics & Gynecology and the American Society of Anesthesiologists state that medically necessary surgery should never be denied regardless of trimester. The level of fetal monitoring depends on whether the fetus is viable at the time of surgery. If the fetus is previable, fetal Doppler heart tones in the preoperative and postoperative areas are sufficient. A viable fetus should, at least, be assessed through fetal heart rate and contraction monitoring preprocedure and postprocedure. Intraoperative fetal monitoring may be considered if the surgical field allows for placement of monitors. The procedure should be performed at a facility with obstetrics, neonatal, and pediatric services readily available. Additionally, the patient should be consented for an emergent cesarean section if necessary [11].

In this case, mediastinoscopy was selected as the initial diagnostic approach due to the anterior and superior mediastinal location of the mass, which appeared to be compressing and displacing the airway. The differential diagnosis included thymoma, lymphoma, and germ cell tumor, and mediastinoscopy was considered the most direct and tissue‐yielding method for obtaining a diagnosis. The decision was made through multidisciplinary consensus between thoracic surgery, oncology, and obstetrics, aiming to achieve adequate sampling while minimizing procedural time and airway manipulation in the setting of pregnancy.

## 4. Conclusion

This case shows the importance of multidisciplinary collaboration between anesthesiologists, obstetricians, and surgeons in managing pregnant patients with high‐risk mediastinal pathology. AFOI remains one of the safest options in securing the airway in patients with AMM. Careful preoperative investigation, intraoperative monitoring, and preparation for hemodynamic instability are critical to prevent vascular or tracheal compression. The successful management of this patient demonstrates the effectiveness of a well‐coordinated approach that prioritizes both maternal and fetal safety.

## Consent

Written informed consent was acquired from the patient whose case details are written in the study, to publish this report in accordance with the journal’s patient consent policy.

## Conflicts of Interest

The authors declare no conflicts of interest.

## Author Contributions

Sabry Ayad: conceptualization; supervision; validation; and writing–review and editing.

Rei Ndoni: conceptualization; writing–original draft; and writing–review and editing.

Aariya Srinivasan: conceptualization; writing–original draft; and writing–review and editing.

Ryan Dougherty: conceptualization; writing–original draft; and writing–review and editing.

Colin Nahrstedt: conceptualization and writing–review and editing.

Jia Liu: Conceptualization.

## Funding

No funding was received for this research.

## Data Availability

The data that support the findings of this study are available from the corresponding author upon reasonable request.
